# *Schmeissneria*: A missing link to angiosperms?

**DOI:** 10.1186/1471-2148-7-14

**Published:** 2007-02-07

**Authors:** Xin Wang, Shuying Duan, Baoyin Geng, Jinzhong Cui, Yong Yang

**Affiliations:** 1 State Key Laboratory of Palaeobiology and Stratigraphy, Nanjing Institute of Geology and Palaeontology, the Chinese Academy of Sciences, 39 Beijing Dong Road, Nanjing 210008, China; 2 Institute of Botany, the Chinese Academy of Sciences, 20 Nanxincun, Xiangshan, Beijing 100093, China

## Abstract

**Background:**

The origin of angiosperms has been under debate since the time of Darwin. While there has been much speculation in past decades about pre-Cretaceous angiosperms, including *Archaefructus*, these reports are controversial. The earliest reliable fossil record of angiosperms remains restricted to the Cretaceous, even though recent molecular phylogenetic studies suggest an origin for angiosperms much earlier than the current fossil record.

**Results:**

In this paper, after careful SEM and light microscopic work, we report fossils with angiospermous traits of the Jurassic age. The fossils were collected from the Haifanggou Formation (middle Jurassic) in western Liaoning, northeast China. They include two female structures and an associated leaf on the same slab. One of the female structures is physically connected to the apex of a short shoot. The female organs are borne in pairs on short peduncles that are arranged along the axis of the female structure. Each of the female organs has a central unit that is surrounded by an envelope with characteristic longitudinal ribs. Each central unit has two locules completely separated by a vertical septum. The apex of the central unit is completely closed. The general morphology places these fossils into the scope of *Schmeissneria*, an early Jurassic genus that was previously attributed to Ginkgoales.

**Conclusion:**

Because the closed carpel is a character only found in angiosperms, the closed apex of the central unit suggests the presence of angiospermy in *Schmeissneria*. This angiospermous trait implies either a Jurassic angiosperm or a new seed plant group parallel to angiosperms and other known seed plants. As an angiosperm, the Liassic age (earliest Jurassic) of *Schmeissneria microstachys *would suggest an origin of angiosperms during the Triassic. Although still uncertain, this could have a great impact on our perspective of the history, diversity and systematics of seed plants and angiosperms.

## Background

Angiosperms are the dominating plant group in current vegetation. They account for the majority of terrestrial primary production, demonstrate the highest diversity in the plant kingdom, and dominate highly diversified habitats [1-4]. The rapid radiation and diversification of angiosperms during the Cretaceous led to major ecological changes on the earth, the latter were a prerequisite for many critical evolutionary events [3] including the later evolution of human beings. However, the origin of this important plant group has remained obscure since the time of Darwin [3,5-8]. Although palaeobotanists have been searching for pre-Cretaceous angiosperms and some speculate that the angiosperm line may extend back to the Triassic [8,9], the earliest angiosperm fossil records are still restricted to the Cretaceous hitherto [3-5,10-12]. Therefore, it is not surprising that whenever something related to the origin of angiosperms is discovered, such as *Sanmiguelia *[13] and *Archaefructus *[6,7,14], it not only receives attention but also triggers controversy within and beyond academic circles. Western Liaoning has been a focus of palaeontological research because of its wealth of fossil plants [7,15-20] and animals [21,22]. Pan's claims of Jurassic angiosperms [15-17] once raised great interest in fossil plants in this region, but they are currently not generally accepted [18,23].

*Schmeissneria *was first identified as a member of the Ginkgoales [24], with a history dating back to 1838 [25]. *Schmeissneria *(*Stachyopitys*) was once thought to be related to conifers [25,26]. Its ginkgoalean affinity was initially proposed based on association: Schenk (1890) classified it as a premature male flower of *Baiera*, therefore placing *Schmeissneria *(*Stachyopitys*) in the Ginkgoales [27]. Based on the data available now, Schenk's conclusion on *Schmeissneria *(*Stachyopitys*) has been proven erroneous. First, the male nature of *Schmeissneria *(*Stachyopitys*) has been disproved [28]. Second, the connection between *Baiera *and *Schmeissneria *has been nullified [24,29]. Third, *Schmeissneria *has been proven connected with *Glossophyllum*? sp. A [30], which is dissimilar to any known ginkgoalean leaf [24]. All of this evidence refuted Schenk's initial proposal on the ginkgoalean affinity of *Schmeissneria *(*Stachyopitys*), and thus *Schmeissneria *lost its affinity. Apparently, Kirchner and Van Konijnenburg-Van Cittert did not realize what they had accomplished and conveniently put *Schmeissneria *in Ginkgoales [24]. In addition, there are a few characters that make *Schmeissneria *even more mysterious: 1) the internal structure of the reproductive organ, which is important for systematics, is hitherto unknown; 2) its connected vegetative parts do not provide enough information to resolve its systematic position; and 3) its winged seeds are never found in other Ginkgoales.

Here new specimens of *Schmeissneria*, *S. sinensis *Wang sp. nov., are reported from the Haifanggou Formation (middle Jurassic) in western Liaoning, China. The new information from these materials allowed us to re-examine the affinity of *Schmeissneria*.

## Results

### Description of the specimens

**Figure 1:**
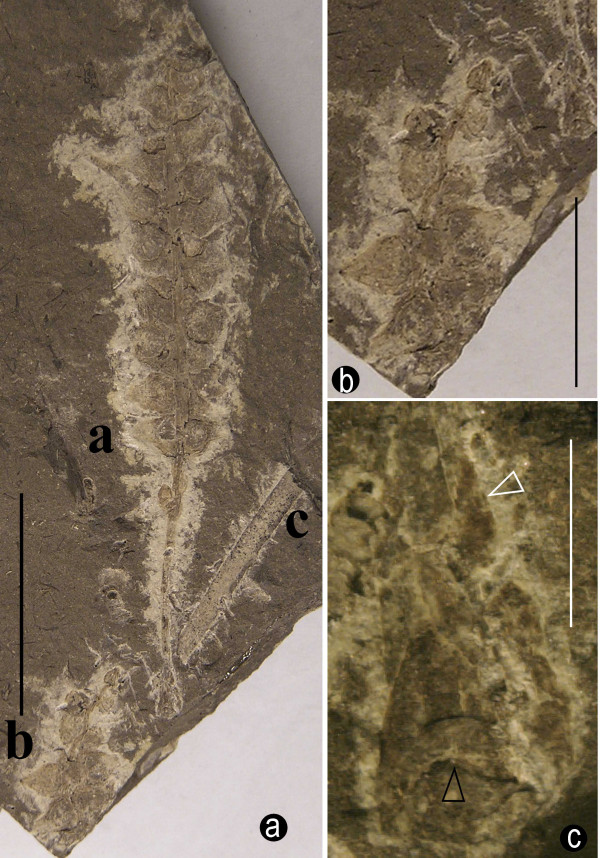
**A general view of female structures, leaf, and short shoot**. a. A general view of two female structures (a, b) and one leaf (c) on the same slab. The specimen A is the holotype, and specimens B and C are the paratypes. Specimen numbers 8604a, 8604b, and 8604c. Bar = 2 cm. b. A detailed view of specimen B in Fig. 1a. Note the twisted axis of the female structure and the attached female organs. Specimen number 8604b. Bar = 1 cm. c. A detailed view of the short shoot. Note the leaf cushion (black arrow) and the axis of the connected female structure (white arrow). Specimen number 8604a. Bar = 2 mm.

A leaf was closely associated with one of the female structures (Fig. 1a). The leaf was incomplete, over 19 mm long and up to 1.8 mm wide (Fig. 1a). It was slender and cuneiform, but its apex was unknown (Fig. 1a). The venation was probably parallel (Fig. 1a). No cuticle could be obtained.

Only the apex of a short shoot was organically connected to a female structure (Figs. 1a,c). It was about 2.4 mm long and 2.3 mm wide, with leaf cushions (Fig. 1c). The leaf cushion was about 0.56 mm high and 1.8 mm wide (Fig. 1c).

**Figure 2:**
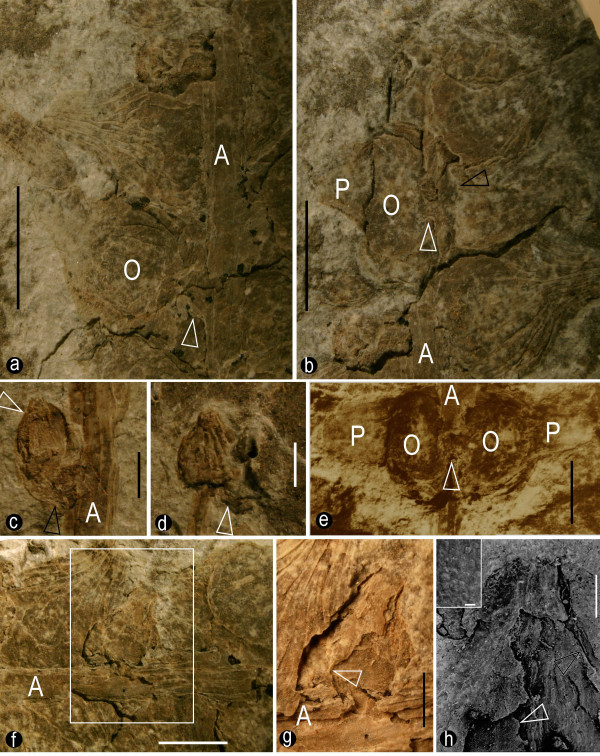
**The morphology and structures of the female organs**. a. Several female organs attached to the axis of the female structure (A). Note the longitudinal ribs on the sheathing envelope and the central unit (O), the short peduncle (arrow), the extended envelope apex, and dark coaly residues. Specimen number 8604a. Bar = 3 mm. b. Several female organs attached to the axis of the female structure (A). Note the envelope (P) and central unit (O), the fused bases of the female organ pair (white arrow), the mark on the central unit left by the fallen envelope (black arrow), and the extended envelope apex. Specimen number 8604a. Bar = 3 mm. c. An isolated female organ pair in the proximal portion of specimen A. Only one of the pair is evident (white arrow); the other one (black arrow) is obscure due to preservation. Note the longitudinal ribs on the axis of the female structure (A) and the less-extended envelope apex. Specimen number 8604a. Bar = 1 mm. d. The top female organ pair in specimen B. Note the longitudinal ribs on the sheathing envelope, the relic of the missing female organ of the pair (arrow), and the less-extended envelope apex. Specimen number 8604b. Bar = 1 mm. e. A female organ pair in the proximal portion of specimen A. Note the axis of the female structure (A), central units (O), sheathing envelope (P), fused female organ bases (arrow), their spatial relationship (the axis of the female structure is in the foreground), and the extended envelope apices. Colored from an original greyscale picture. Refer to Fig. 4a. Specimen number 8604a. Bar = 2 mm. f. Female organs attached to the axis of the female structure. Note the longitudinal ribs on the axis of the female structure (A), female organs of various sizes and orientations, longitudinal ribs on the envelope, and the extended envelope apex. Specimen number 8604a. Bar = 2 mm. g. A detailed view of the rectangular region in Fig. 2f. Note the exposed internal details of the central unit, smooth wall in the lower part, rough wall in the upper part, large locule, and dark coaly residue. Because the septum and part of the central unit are raised above the level of the side wall, they cast a dark shadow on the latter (arrow) and the shadow extends from the base to the upper parts of the central unit. Refer to Fig. 4b. Specimen number 8604a. Bar = 1 mm. h. A view of a female organ with the exposed interior details of the central unit with the SEM. Note the central unit margin (black arrow) and papillate septum (white arrow and upper left inset) distinct from the interior wall with longitudinal ribs. Specimen number 8604a. Inset bar = 20 μm, Bar = 0.5 mm.

The female structures were spicate, up to 9.4 mm wide, at least 6 cm (Fig. 1a) and 1.8 cm (Fig. 1b) long, respectively and generally tapering apically (Figs. 1a–b, 4d). The axes of the female structures were up to 1.3 mm across basally and only 0.2 mm across apically (Figs. 1a–c, 2a–d). The structures were either straight (Fig. 1a) or sinuous (Fig. 1b), longitudinally ribbed (Figs. 2a–c,f, 4d), but free of female organ for about 1.8 cm at the base (Figs. 1a, 4d). One of the female structures was organically connected to the apex of a short shoot (Figs. 1a,c). A female structure had more than 21 female organs attached (Fig. 1a). Generally, the female organs at the basal part were larger and more mature than those at the distal (Figs. 1a–b), except for an isolated female organ pair at the base (Figs. 1a, 2c, 4d). At least some female organs were connated basally (Figs. 2b–e, 4a,d). The peduncle of the female organ pair, rarely seen, was about 0.5 mm long (Fig. 2a).

**Figure 3:**
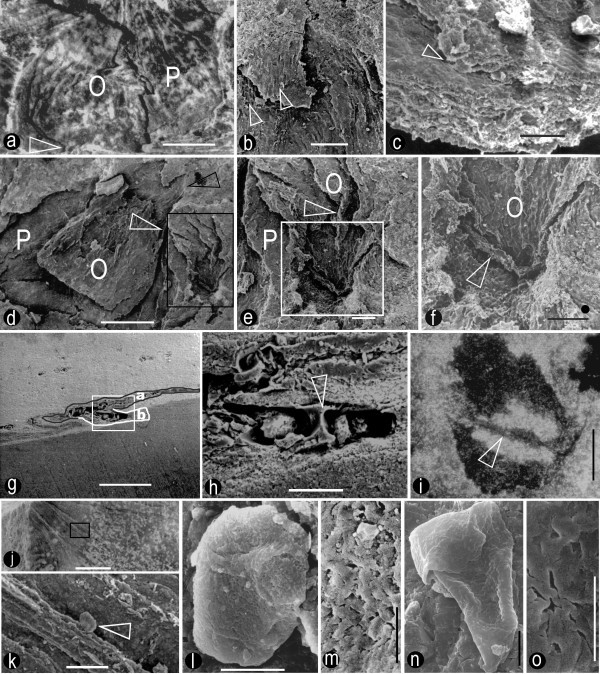
**Detailed views of the internal structure of female organs**. a. A female organ with a broken envelope and its exposed central unit. Note the sheathing envelope (P), central unit (O), and longitudinal ribs with dark coaly residues on them. The central unit is attached to the receptacle by a wide base (arrow). Specimen number 8604a. Bar = 1 mm. b. A view of the envelope apex of the female organ in Fig. 3d with the SEM. Note the elongated cells on the smooth epidermis and slightly elongated cells in the tissue of the envelope (arrows). Specimen number 8604b. Bar = 0.1 mm. c. A view of an envelope fragment with the SEM. Note the smooth surfaces and the border between different parts of the envelope (arrow). Specimen number 8604a. Bar = 50 μm. d. A view of a female organ with the envelope (P) and the central unit (O) with the SEM. The texture and the central unit outline (white arrow) could be traced to the envelope apex (black arrow). On the right, there is another female organ with its apex plunging into the sediment matrix (black rectangle). Specimen number 8604b. Bar = 0.5 mm. e. A detailed view of the rectangular region in Fig. 3d with the SEM. Note the spatial relationship between the envelope (P) and the central unit (O), and the longitudinal ribs (arrow) on the internal walls of the central unit. Specimen number 8604b. Bar = 0.1 mm. f. A detailed view of the apex of the central unit in Fig. 3e (rectangle) with the SEM. Note the septum (arrow) across the central unit (O) apex. The black dot beside the bar is about 20 μm in diameter, the size of an average pollen grain. An entry point for a pollen grain of similar size, if present, would be hard to ignore in this image. Therefore, at least the tip of the upper locule (carpel), which is not eclipsed by the septum vestige or in its shadow, is closed. Specimen number 8604b. Bar = 0.1 mm. g. A cross section of two female organs embedded in the sediment. Note the pale sediment (upper half), dark resin (lower half), and darker stripes of two fused female organs (a and b, outlined by black and white lines, respectively). Specimen number 8604a. Bar = 0.5 mm. h. A detailed view of the female organ in Fig. 3g. Note the septum (arrow) and its smooth connection to the side walls of the central unit. Specimen number 8604a. Bar = 0.1 mm. i. A thin section across the apex of the central unit in Fig. 3f. Note the septum (arrow) separating two locules and its smooth connection to the side walls. Light microscope. Specimen number 8604b. Bar = 0.1 mm. j. A view of the internal surface of an envelope apex with the SEM. Note the converging longitudinal ribs. Specimen number 8604a. Bar = 0.5 mm. k. A detailed view of the rectangular region in Fig. 3j. Note the pollen grain (arrow) adherent to one of the longitudinal ribs on the internal surface of the envelope. Specimen number 8604a. Bar = 50 μm. l. A detailed view of the pollen grain in Fig. 3k. Specimen number 8604a. Bar = 10 μm. m. The rugulate sculpture on the pollen grain in Fig. 3l. Specimen number 8604a. Bar = 2 μm. n. Another pollen grain adherent to the internal surface of the envelope apex. Specimen number 8604a. Bar = 5 μm. o. The rugulate sculpture on the pollen grain in Fig. 3n. Specimen number 8604a. Bar = 2 μm.

**Figure 4:**
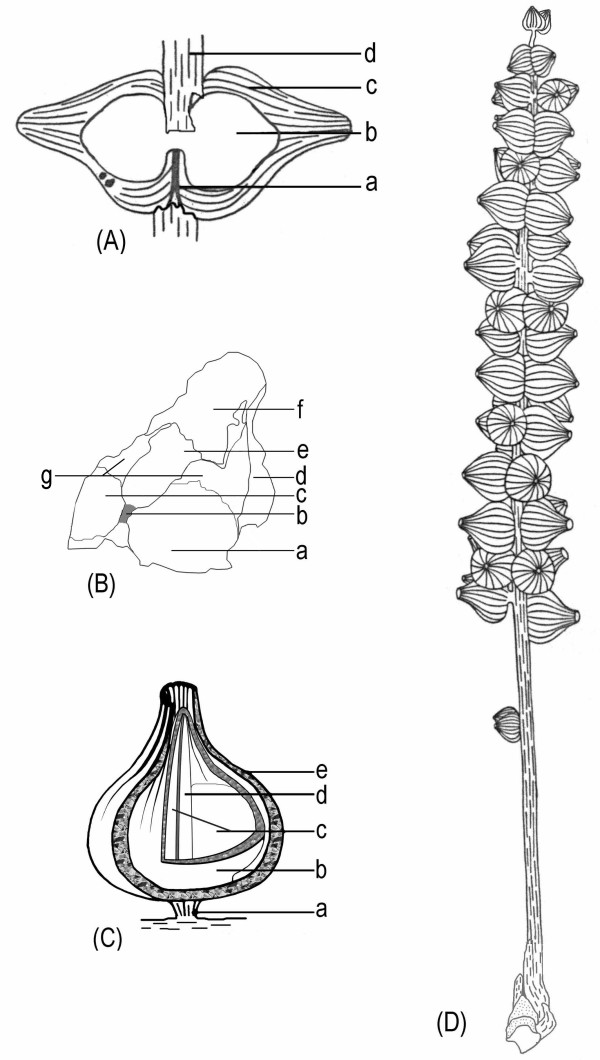
**Diagrams of the female organs and the reconstructions of *S. sinensis***. a. Schematic diagram of the female organ pair shown in Fig. 2e. Note the female organs' fused bases (a), the central unit (b), the sheathing envelope (c), and the axis of the female structure (d). b. Schematic diagram of the female organ in Fig. 2g. Note the smooth locule walls (a and c, probably due to the fallen ovules) and the rough internal walls (g) on each side of the septum (b), different parts of the envelope (d and f), the relic of the broken central unit (e), the vertical septum (b) connected to the base of the central unit. c. A reconstruction of a female organ of *S. sinensis*. Note the short peduncle of the female organ pair (a) connected to the axis of the female structure (for simplicity, only one organ of the female organ pair is shown here), the central unit (b), two locules (c), the septum (d) separating the two locules, and the sheathing envelope (e). d. Schematic diagram of *S. sinensis*. From the bottom, note the apex of the short shoot, axis of the female structure, isolated immature female organ pair, clustered female organ pairs along the axis of the female structure, and terminal female organ pair.

The female organs were about 1.6–4.6 mm long, about 1.2–4 mm in diameter, widest at the base and constricted at the apex (Figs. 2a–f, 3a, 4a,c). They had an onion-like appearance, including a central unit and a sheathing envelope (Figs. 2b,e, 3a,d, 4c,d). The envelope was longitudinally ribbed internally and externally (Figs. 2a–b,f, 3a,j, 4a,c,d). The apices of the female organs pointed away from the axis of the female structure (Figs. 2a–b, 4a,d). The envelope apex of a large female organ (Figs. 2a–b, 2e–f) was more extended than that of a smaller one (Figs. 2c–d, 3d). The envelope, sheathing around the central unit, was of multiple parts (Fig. 3c), and inflated (Figs. 2a–b, 3a). The surface of the envelope was smooth locally with elongated epidermal cells (Fig. 3b). The cells within the envelope were elongated: 18–33 μm long and 6–12 μm wide (Fig. 3b). The rugulate pollen grains found on the internal surface of the envelope apex were about 26 μm in diameter (Figs. 3j–o). The central units were about 1.5 to 3.3 mm long and 1 to 3.2 mm in diameter, widest basally and constricted apically (Figs. 2a–b,e, 3a,d, 4a,c). The central unit was attached to a receptacle by a wide base about 1.6 mm in diameter (Fig. 3a), with its apex approaching that of the envelope (Figs. 3d–f, 4c). The distal part of the central unit wall was longitudinally ribbed internally and externally (Figs. 3a,e). No elaborated pollen reception site was observed (Figs. 3d–f,i). The central unit was bilocular, completely separated by a 9 to 19 μm thick vertical septum (Figs. 2f–h, 3d–i, 4b–c). The central unit internal walls were smooth (Figs. 2g, 4b) and flat at the base and rough in the upper part (Figs. 2g, 3e,f, 4b). The septum was complete, extending from the base (Figs. 2g, 4b), through the middle part (Figs. 3g–h) to the apex (Figs. 2h, 3e–f,i, 4c) of the central unit. The septum was papillate (Fig. 2h and its inset).

**Remarks: **Although two rugulate pollen grains (Figs. 3j–o) were found on the internal surface of the envelope apex, their relationship to *S. sinensis *was tentative and subject to further validation. *Stachyopitys preslii *(possible male organ of *Schmeissneria *[24]) was only associated with but never connected to *Schmeissneria microstachys *[24], and may also be related to other Ginkgoalean plants [24].

## Discussion

The specimens studied here were generally identical to *Schmeissneria *[24], especially the holotype of *S. microstachys *(pl. I, fig. 1) [24]. The latter closely resembled *S. sinensis *in spicate structure, paired female organs, female organ morphology, longitudinally ribbed axes of female structures, and the insertion of a female structure on the apex of a short shoot, even though the specimens described here and *S. microstachys *were from different continents and geological epochs, and *S. sinensis *had more densely clustered female organs and much shorter peduncles of female organ pairs (0.5 mm rather than 2 mm long). Fortunately, the internal structure was preserved in *S. sinensis *and thus shed new light on its affinity.

### Structure and Interpretation

In all non-angiospermous seed plants, there is an opening at the nucellar apices to allow the pollen grains to approach the ovules [31,32]. The dimension of the opening has to be large enough to let pollen grains pass through. Unlike seed ferns or other gymnosperms, no opening was found at the apex of the central unit in *S. sinensis *(Figs. 3d–f). In the view from the interior of the central unit to its apex (Fig. 3f), any opening larger than 20 μm (the normal size of an average pollen grain) should be visible if present on the central unit wall. Although the preservation of the fossils reported here was not perfect, the female organs had both the cellular details (Fig. 3b) and a septum only about 10 μm thick (Fig. 3i) preserved. The preservation of such fine details suggested that preservation fidelity was high enough for structures above the cellular level. Therefore the central unit apex of *S. sinensis *was physically closed, at least to pollen grains. This was essentially different from the situation in *Caytonia*, in which there are numerous ovules within a cupule that has an opening for pollen grain entry before pollination [33,34]. There were two alternative interpretations for the absence of pollen entry in *Schmeissneria*: one was that the pollen entry did not exist at all; the other was that the pollen entry had been obliterated. The latter situation has been seen in *Gnetum *[32,35], *Ephedra*, *Pinus*, *Cedrus*, *Cephalotaxus *[36], and possibly in *Caytonia *[33,34], in all of which the pollen canals were plugged or obliterated by tissue outgrowth or cell proliferation after pollination. This tissue growth or cell proliferation is concomitant with morphological changes [35,36]. However, in the case of *Schmeissneria *this alternative appeared unlikely for the following reasons: 1) the female organs in Figs. 3d–f were in their early stages (pre-pollination), suggested by their smaller sizes and morphology different from winged seeds [24]; 2) there was neither any trace of abnormal tissue outgrowth nor sudden changes in the wall at the apex of the central unit (Figs. 3d–f), unlike what is observed in *Gnetum *[35], *Cephalotaxus*, and *Ephedra *[36]. Consequently, the other alternative was more reasonable and acceptable. This was the major reason that the authors correlated the structures of *Schmeissneria*'s central unit, envelope, female organ and female structure with an angiosperm's gynoecium, perianth, flower and inflorescence.

A septum extended from the base (Figs. 2g, 4b), through the middle (Figs. 3g–h), to the apex (Figs. 2h, 3e,f,i, 4c) of the central unit. The septum separated the central unit into two independent locules (Figs. 2f–h, 3h–i, 4b–c). The latter were suggestive of two carpels in a central unit (equivalent to gynoecium in angiosperm) [37]. The septum appeared as a ridge raised above the smooth wall of the central unit in the longitudinal view (Fig. 2g). Its smooth connections to the side walls (Figs. 3h–i) and base (Fig. 2g) of the central units suggested that the septum represented an original structure rather than an artefact or alteration. The repeated presence of a septum of various poses in four individual female organs (Figs. 2g–h, 3d–i) also strongly suggested its truthful existence. Up until now there has been no report of a complete septum in a seed or ovule in any gymnosperm. Sometimes paired ovules in *Ginkgo *may appear to have a membraneous division in between. However, their ovules have never been completely enclosed before pollination, the tips of the paired ovules point to different directions, and there is no empty interior space within any ovule. Therefore the paired ovules of *Ginkgo *were distinct from the central unit of *Schmeissneria*.

*Schmeissneria sinensis *had female organs of various ontogenetic stages, from small premature ones at the top to large mature ones at the base of the female structures (Figs. 1, 2, 3, 4). Considering the slight morphological difference between the topmost (most immature) and bottommost (most mature) female organs, it was reasonable to assume that the female organs reported here, especially the smaller ones, were not yet pollinated. The rough internal surface in the upper portion of the central unit (Fig. 2g and Fig. 4b(g)) and the longitudinal ribs on it (Fig. 3e), in contrast to the smooth surface in the lower portion of the internal surface of the central unit (Fig. 2g and Fig. 4b(a,c)), suggested that the upper portion of the central unit was empty, while the lower part was occupied by another inherent substructure (probably an ovule). This situation was unlikely in ovules or seeds, which rarely, if ever, have an empty interior space. It was also unlikely to be a result of differentiated preservation related to histological differences because the presence or absence of tissues is a morphological rather than histological character. The winged seeds connected to vegetative parts reported by Kirchner and Van Konijnenburg-Van Cittert (Fig. 1b, [24]) were distinct from the female organs reported here, implying the immaturity of *S. sinensis*. Considering the aforementioned details, since Wcislo-Luraniec [28] and Kirchner and Van Konijnenburg-Van Cittert [24] have proven the female nature of *Schmeissneria*, the immature central unit with two locules and a closed apex sheathed by an envelope can be interpreted as a gynoecium of two carpels surrounded by a perianth. This suggested that the carpels (gynoecium) in *Schmeissneria *were closed before pollination, a situation quite different from the "angiospermy" after pollination in gymnosperms [38].

### Affinity

In 1838, Presl (pl. 33, fig. 12 only) reported fructifications from Keuper Sandstone (actually Liassic age [24]) of Reundorf near Bamberg, Germany [25]. He considered them male flowers of *Pinites microstachys *(Conifers). Schenk (pl. XLIV, figs. 11, 12 only, 1867) investigated similar fossils from Veitlahm near Kulmbach, describing them as female flowers of *Stachyopitys preslii*, which was associated with *Schizolepis *(a coniferous genus) [26]. Later Schenk (fig. 180b only, 1890) assigned *Stachyopitys preslii *as male fructifications of *Baiera münsteriana *(Ginkgoales), and interpreted the formerly "female fructifications" as male flowers in an early stage [27]. This association between *Baiera *and *Stachyopitys *proposed by Schenk [27] was later widely accepted (taf. 29, abb. 4 [39]; fig. 239c [40]; fig. 375d [41]; abb. 303h [42]; fig. 198B [31]; taf. 1, fig. lower left [43]; fig. 35d [32]). Actually, fossils of various affinities had been lumped into *Stachyopitys preslii *[44]. Wcislo-Luraniec (pl. 1 and textfigs. 1, 2, 1992) cast doubt on the male nature of *Stachyopitys preslii *because of the discovery of "cupules," thereby proving the female nature of the fossil [28]. At the same time, *Stachyopitys preslii *was found connected with Weber's (p58, taf. 5, fig. 51 [30]) *Glossophyllum*? sp. A [29]. With more complete fossil materials available, a new genus, *Schmeissneria *(Ginkgoales), was established for the female *Stachyopitys *and officially separated from the male *Stachyopitys *(Figure 1 and pls. I-III, pl. IV, fig. 1, [24]). It appeared that Kirchner and Van Konijnenburg-Van Cittert did not realize that they had undermined the ground for the ginkgoalean affinity of the fossil and uncritically accepted the previous conclusion as true [24]. Furthermore, they did not give any valid reason for why they put the genus in Ginkgoales except for comparing the paired "ovules" of *Schmeissneria *and the paired ovules of *Ginkgo *in two sentences (p.207) [24]. Apparently, their comparison was not sufficient to place the fossil in Ginkgoales with confidence because the characteristic collar at the base of *Ginkgo *seed was absent in all specimens of *Schmeissneria *and the winged seeds of *Schmeissneria *were never seen in any Ginkgoales. However, re-examining the references indicated that 1) the discovery of the connection between *Stachyopitys *(*Schmeissneria*) *preslii *and Weber's *Glossophyllum*? sp. A disproved the relationship between *Stachyopitys *(*Schmeissneria*) *preslii *and *Baiera münsteriana *proposed by Schenk [27]; and 2) *Glossophyllum*? sp. A did not belong to the ginkgoalean *Glossophyllum *[24]. Therefore, *Schmeissneria *was dangling phylogenetically. Since the connected vegetative organs could not resolve its affinity, the affinity of *Schmeissneria *had to be resolved based on the internal structure of its own female reproductive organs.

Seed plants include two major groups, gymnosperms and angiosperms. Currently the known major Mesozoic and extant gymnospermous groups include Ginkgoales, Cycadales, Bennettitales, Coniferales, Glossopteridales, Caytoniales, and Gnetales. Among them, Cycadales have pinnate leaves and ovules/seeds attached to the margins of megasporophylls [5,31,32]; Bennettitales have pinnate leaves and conspicuous cone-like reproductive organs with ovules/seeds arranged on a central receptacle [5,31,32]; Coniferales have needle-like or various leaves and more or less cone-like structures composed of bract-scale complexes, except for all or some elements of Taxaceae and Podocarpaceae [5,31,32,45], the latter two are distinctly different from *Schmeissneria*; Glossopteridales have reticulate leaves and their megasporophylls adnated to the adaxial of the foliage [5,31,32,46]; Caytoniales have reticulate leaves and cupules with inverted openings oppositely arranged along the laterals of the megasporophyll axis [5,32]; and Gnetales have a micropylar tube, articulate shoot, opposite/whorled leaves, opposite/whorled bracts in the reproductive cone, ovuliferous units in the bract axil [31,32,47,48] (Table 1). These characteristics clearly distinguished these groups from *Schmeissneria*. Therefore these groups will not be considered further in the following discussion on the affinity of *Schmeissneria*, and the only possibilities remaining are Ginkgoales, Angiosperms, or a new group of seed plants.

**Table 1:** Comparison of *Schmeissneria *to Ginkgoales, Gnetales, and angiosperms.

Characters	*Schmeissneria *[24,*]	Ginkgoales[24,31,32,45]	Gnetales[31-32,47,83-84]	Angiosperms[1]
Long/short shoot	+	+	-	+/-
Shoot articulate	-	-	+	+/-
Phyllotaxis	Irregular spiral	Spiral on short shoot	Opposite/whorled	Opposite, whorled, spiral
Leaf	Slender-cuneiform, obtuse apex	Fan-shaped, or cuneiform	Strap-like, triangular, linear or oval-shaped	Various
Venation	Parallel	Dichotomous	Parallel or reticulate	Various
Unisexuality	+	+	+ (-)	+/-
Reproductive axis articulate	-	-	+	+/-
Bract	-	-	+	+/- (?)
Bract arrangement	N/A	-(? bract scale seed complex)	Whorled/paired	Whorled, paired, or N/A
Ovuliferous unit position	On peduncle tip, in pair	On peduncle tip, single, pair or more in group	In bract axil, one to many in a group	Terminal/axillary
Ovuliferous unit with septum	+	-	-	+/-
Ovule	Completely enclosed by central unit wall	Exposed through micropyle	Exposed through micropylar tube	Completely enclosed by carpel or secretion
Micropylar tube	-	-	+	-

Note that the character of enclosed ovule distinguishes *Schmeissneria *and angiosperms from the other two groups.*: information from this paper.

Several characters are used to distinguish angiosperms and gymnosperms, including enclosed ovules/seeds, double fertilization, vessel elements, reticulate venation, and tectate-columellate pollen wall structure. However, it is harder to draw a line between these two groups than it might appear: neither of the above characters is a touchstone for angiosperms [1,3]. None of the above characters is unique to angiosperms. All the ovules in gymnosperms are exposed when pollination occurs, but at least some of the ovules/seeds are enclosed after fertilization (Caytoniales [33]; Gnetales [35]; Coniferales and Gnetales [36]; Coniferales [38]). Angiosperms have completely closed carpels. However, in some basal groups this closure is by secretion, and not by postgenital fusion (*e.g.*, Amborellaceae, Schisandraceae, Austrobaileyaceae, Trimeniaceae [49]). Double fertilization has been reported in non-angiosperms, as in *Ephedra *[31,50] and *Abies *[31]. Vessel elements are also present in various non-angiosperms, such as *Selaginella*, *Equisetum*, *Pteridium *[1], Gigantopteriales [51], and Gnetales [1,4,31,32]. Reticulate venation has been reported in non-angiosperms, including Dipteridaceae [52-55], Gigantopteriales [56,57], Caytoniales [33,58], Glossopteridales [5,40,46,58], Bennettitales [32], and Gnetales [31,32]. Pollen grains with an angiosperm-like wall structure have been reported in many pre-Cretaceous plants [59-61] that are regarded as non-angiosperms by others [62]. According to Tomlinson and Takaso [38], the only consistent difference between angiosperms and gymnosperms is that the ovules at pollination are exposed in gymnosperms, but enclosed in angiosperms. Fortunately, the closed carpel at or before pollination is a character that is sufficient to identify an angiosperm alone. This was one of the characters used here to resolve the affinity of *Schmeissneria*.

Two characters separated *Schmeissneria *from known gymnosperms: the vertical complete septum and the closed apex of the central unit. Considering all available information and the definitions of plant groups, there were two alternatives left to us: 1) accepting that *Schmeissneria *as a new angiosperm in the Jurassic, or 2) proposing *Schmeissneria *as a new gymnosperm. Although the specialized features of the early Cretaceous angiosperm *Archaefructus *[14] and other data [8,9] may imply the possible existence of angiosperms before the Cretaceous, *Schmeissneria *did not look like any known typical angiosperm. However, this dissimilarity was conceivable and understandable since 1) "angiophytes" had not evolved any typical identifiable angiospermous character [8], and 2) many angiospermous taxa were much more diversified then and much of that diversification had since become extinct [62]. Extreme caution should be exercised when a Jurassic angiosperm, along with their relatives of which we know very little, is compared with the extant angiosperms.

If accepted as an angiosperm, because of its early Jurassic age in Europe, *Schmeissneria *would push the origin of angiospermy back to the Triassic. This would make the claims of Triassic angiosperms [13,59-61], [63,64] less surprising, and also help to bridge the gap between the fossil record [3,5,10-12,65] and molecular data [66-72]. However, it should be kept in mind that *Schmeissneria *might well represent early angiosperms still sporadic in the vegetation dominated by gymnosperms, that it might bear no direct relationship with any known angiosperm, and that the presence of *Schmeissneria *in the flora was still far different from the radiation and diversification of angiosperms.

## Conclusion

*Schmeissneria *is an interesting Jurassic plant that bears a trait of angiosperms (two separated locules in a central unit with a closed apex). This feature has a counterpart in angiosperms (two carpels in the gynoecium). Based on the current definition of angiosperms, *Schmeissneria *could be classified as an angiosperm. Otherwise, a new group, Schmeissneriales, would have to be established for *Schmeissneria*. The bottom line is that in whatever position *Schmeissneria *is placed in the future, it increases the diversity of seed plants and challenges people regarding the systematics of seed plants. Undoubtedly, *Schmeissneria *requires further study.

### Systematics

*Schmeissneria *Kirchner et Van Konijnenburg-Van Cittert 1994, emend.

**Type species**: *Schmeissneria microstachys *(Presl 1838) Kirchner and Van Konijnenburg-Van Cittert 1994.

**Emended diagnosis **(based on previous work and present observation)

Plants with long- and short-shoots. Leaves inserted helically on short shoots. Short shoots with leaf cushions. Leaf slender, slightly cuneiform, apex obtuse. Veins parallel, more than two in the proximal part of the leaf, branching in the lower third of the lamina.

Female structures spicate, with a slender axis. Axis of the female structure longitudinally ribbed. Female organ pairs connated basally, borne on a peduncle, arranged along the axis of the female structure. Female organ with a central unit and a sheathing envelope. Envelope of undetermined number of parts, inflated, longitudinally ribbed internally and externally. Central unit bilocular, astylous, with a vertical septum, with longitudinal ribs distally both internally and externally. Seed winged (?).

**Type locality**: Reundorf near Bamberg, Germany.

**Further locality**: Oberwaiz, Unternschreez (Lautner) and Schnabelwaid (Creußen) near Bayreuth, Veitlahm, Pechgraben near Kulmbach, Großbellhofen, Rollhofen (Wolfshöhe) northeast of Nuremberg (all in Germany); Odrowaz, Holy Cross Mounts, Poland; western Liaoning, China.

**Stratigraphic horizon**: Liassic, lower Jurassic (Germany and Poland); middle Jurassic (China).

**Remarks**: There are a few fossil taxa similar to *Schmeissneria*, including *Ktalenia*, *Schizolepis*, *Drepanolepis*, *Caytonia*, *Leptostrobus*, and *Karkenia*. Among them, *Schizolepis *has spirally arranged bilobate two-seed-bearing scales in bract axils [73]; *Drepanolepis *has spirally arranged sickle-shaped appendages bearing a single seed [74]; *Ktalenia *has oppositely arranged globose seed-bearing cupules with micropyle pointing downward [75]; *Caytonia *has oppositely arranged globose multiple-seed-bearing cupules with micropyles pointing to the axis [33,58]; *Leptostrobus *has spirally arranged bivalvate multiple-seed-bearing cupules in which the two valves share a common slit-like opening pointing outward [76,44]; and *Karkenia *is an oval-elongate fructification of irregularly disposed atropous, pedunculate ovules/seeds with micropyle pointing to the axis [24,77,78], a genus distinctly different from *Schmeissneria *[24]. These characters distinguish these genera from *Schmeissneria*, which has paired female organs on a peduncle that are arranged along the axis of the female structure [24, also in this paper] (for details, see Table 2).

**Table 2:** Comparison between *Schmeissneria *and other possibly related fossil taxa.

	Appendage arrangement	Appendage shape	No. appendages per peduncle	Appendage opening orientation	Appendage apex	Seed/central unit position	Ovule completely enclosed	Vertical septum	No. ovules/seeds per cupule/central unit	Peduncle connects to
*Schmeissneria*(young) [24,*]	Irregular	Globose, in pair	2	Away from axis	More or less extended	Within the sheathing envelope	+	+	2?	Envelope
*Schmeissneria*(mature)[24]	Irregular	Sickle-like, single	1–2	Away from axis	Wing-shaped	Below the wing, enclosed (?)	?	?	1?	Seed?
*Ktalenia*[75]	Opposite, subopposite	Globose	1	Down	More or less extended	Within cupule	-	-	1–2	Cupule
*Schizolepis*[73]	Spiral	Bilobate	1	Up	Lobate	Axil of scale	-	-	2	Scale
*Drepanolepis*[74]	Spiral	Sickle-like	1	Up	Wing-shaped	Axil of scale	-	-	1	Scale
*Leptostrobus*[44,76]	Spiral	Round bivalvate	1 (2 valves)	Away from axis	Round, thickened margin	Within capsule, axil of valves	-	-	Several	Cupule
*Caytonia*[33,58]	Opposite	Globose	1	To axis	Extended lip	Within capsule	-	-	Several	Cupule
*Karkenia*[24,77-78]	Irregular	Round-oval	1	To axis	Acuminate apex	On peduncle	-	-	1	Ovule/seed

Note the characters (enclosed ovule, vertical septum) distinguishing *Schmeissneria *from the others. *: information from this paper.

*Schmeissneria sinensis *Wang sp. nov.

**Diagnosis**: The same as that of the genus, except that female organs are densely clustered along the axis of the female structure and the peduncles of the female organ pairs are short in this new species.

**Description **(see Results)

**Holotype**: 8604a.

**Paratype**: 8604b, 8604c.

**Etymology**: *sinensis *referring to China, for specimens found in China.

**Type locality**: Sanjiaochengcun, Jinxi, western Liaoning, China.

**Stratigraphic horizon**: the Haifanggou Formation, middle Jurassic; equivalent to Aalenian-Bajocian.

**Depository**: Institute of Botany, the Chinese Academy of Sciences, Beijing, China.

**Remarks**: The specimens described here and *S. microstachys *were from different continents and geological epochs, and *S. sinensis *had more densely clustered female organs and much shorter peduncles of female organ pairs (0.5 mm rather than 2 mm long). Therefore, a new species, *S. sinensis*, was established for the specimens from Liaoning, China.

## Methods

**Figure 5:**
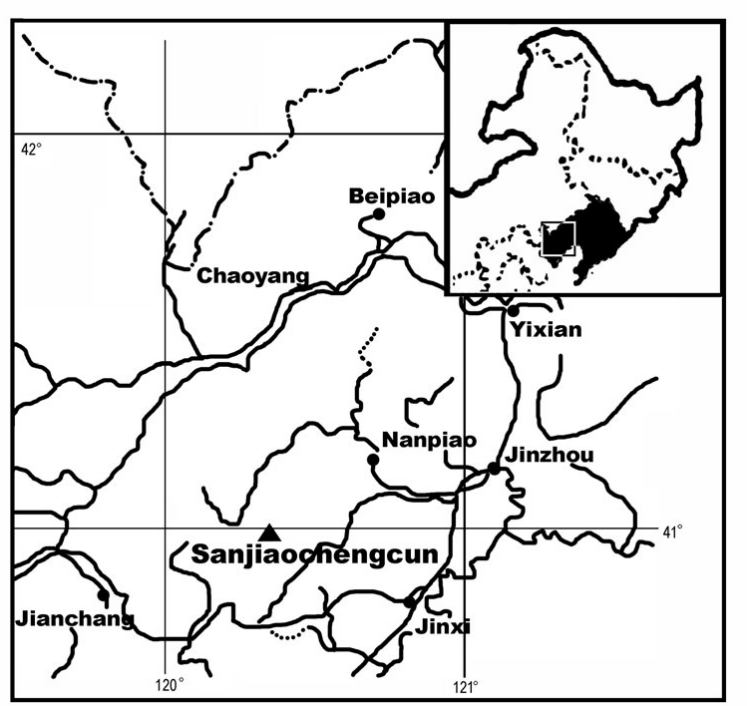
**The geographic position of the type fossil locality of *S. sinensis***. The upper right inset shows northeast China, and the black area within is Liaoning Province. The rectangular area in the inset is shown in detail in the main map. The black dots are the major cities in the region, and the black triangle is the type locality of *S. sinensis*, Sanjiaochengcun, Jinxi, Liaoning, China (120°21'E, 40°58'N).

The specimens were collected from Sanjiaochengcun (120°21'E, 40°58'N), Jinxi, Liaoning, China (Fig. 5) in 1988. The bed yielding the present materials belongs to the Haifanggou Formation [15-17,19]. Most people think that the age of the Formation is middle Jurassic [15-17,19,79-81]. The flora of the Haifanggou Formation (including 122 species in 48 genera, dominated by a *Coniopteris*-*Phoenicopsis *assemblage) iscomparable to the Yorkshire flora (middle Jurassic) of England [79]. This conclusion is supported by chronostratigraphy and other fossils, including ostracodes, bivalves, insects, vertebrates, and palynology [79]. Based on K-Ar, ^40^Ar-^39^Ar, Rb-Sr, U-Pb, and Sm-Nd datings, the volcanic rocks of the Lanqi Formation (the formation just above the Haifanggou Formation) in Liaoning are 160 to 170 Ma old [79]. Thus, under any circumstance, *S. sinensis *found in the Haifanggou Formation is older than the Lanqi Formation [79] and at least 160 Ma old. The latest dating, based on volcanic rock and biota, of the Daohugou Formation (equivalent to the Haifanggou Formation) is Callovian to Oxfordian (155 to 165 Ma) [82]. Furthermore, the European *Schmeissneria *are from the Triassic-Jurassic boundary (Lias α [24]; lower Lias [28]). Considering all the evidence, the authors accept the age of *S. sinensis *as middle Jurassic.

The studied specimens included two weakly coalified compressions of female structures and one associated leaf. Coaly membranes were found only on the surface of the specimens (Fig. 3a), while most parts of the specimens were replaced by unidentified minerals. The fossils were exposed by trimming away the fine matrix. They were observed under a Zeiss stereomicroscope, photographed with a Nikon 4500 and a Nikon Stereomicroscope SMZ1000 with digital camera DMX1200F. Some of the specimens (Figs. 3b–f, 3j–o) were coated with gold and observed under a Hitachi S800 SEM at Institute of Botany, the Chinese Academy of Sciences. After SEM observations, a piece of specimen (Fig. 3d–f) was embedded in an Epon 812 resin, ground into a thin section, and observed with the light microscope (Fig. 3i). One of the trimmed fragments was embedded in Epon 812 resin, ground, coated with gold, and observed with the SEM again (Figs. 3g–h). The SEM images were recorded on black-white negatives. All photographs were later scanned, processed, and pieced together for publication using Photoshop 7.0.

## Authors' contributions

XW carried out the fossil processing, photography, SEM, figure preparation, data analysis and interpretation, manuscript drafting and finalization. SD & BG did the field work, collected the specimen, participated in the data analysis and interpretation, and manuscript modification. JC participated in the data analysis and interpretation, and manuscript modification. YY participated in the data analysis and interpretation, and manuscript modification. All authors read and approved the final manuscript.
